# Preparation of Rice Bran-Enriched Sweet Rice Wine and Its Quality Improvement Through Extrusion

**DOI:** 10.3390/foods14091582

**Published:** 2025-04-30

**Authors:** Wantian Li, Teng Han, Yuxin Wen, Hao Zhang, Dandan Li

**Affiliations:** College of Food Science and Technology, Nanjing Agricultural University, Nanjing 210095, China

**Keywords:** extrusion, rice bran, sweet rice wine

## Abstract

This study aimed to improve the quality of sweet rice wine through exogenous addition of rice bran and extrusion. The effects of directly fermenting brown rice and adding rice bran on key quality indicators including alcohol content, sugar-to-acid ratio, antioxidant properties, and sensory characteristics were evaluated. Compared to direct fermentation, the exogenous addition of rice bran significantly improved overall wine quality, with 4% rice bran yielding the optimal alcohol content of 1.6% vol and sugar-to-acid ratio exceeding 40. Extrusion modified the physical structure of rice bran, improving its performance as a fermentation substrate. Consequently, both the antioxidant activity and flavor profile of the sweet rice wine were further enhanced. These findings provide valuable theoretical insights for the development of high-quality whole-grain foods.

## 1. Introduction

Sweet rice wine, also known as *Jiuniang* or *tianjiu*, is a traditional Chinese fermented rice product produced through solid-state fermentation using sweet-type starter cultures, with *Rhizopus oryzae* as the primary microorganism [1,2]. During fermentation, *Rhizopus oryzae* simultaneously performs saccharification and fermentation, converting starch into sugars and subsequently into alcohol. This process results in a particularly pleasant and palatable alcohol beverage characterized by a pronounced sweetness, mild alcohol flavor, and a distinctive rice aroma [3]. Various studies have demonstrated that sweet rice wine is rich in bioactive compounds, including polyphenols, polysaccharides, oligosaccharides, peptides, organic acids, and trace elements [4,5]. These components contribute to a variety of health-promoting effects, such as antioxidation, blood glucose regulation, lipid reduction, blood pressure lowering, immune enhancement, and gut health improvement [4]. Given the growing consumer interest in health, sweet rice wine aligns well with modern dietary preferences and holds significant market potential.

The traditional process of sweet rice wine production primarily uses polished glutinous rice, rich in amylopectin, as the main raw material [5]. This process involves steps such as washing and soaking, steaming and cooling, mixing with starter cultures, and fermentation [5]. Due to the highly branched structure of amylopectin, glutinous rice gelatinizes easily during steaming [6] and is readily hydrolyzed into monosaccharides and dextrin by amylases during fermentation [7], thus contributing to the production of rice wine with a rich aroma and a smooth taste. Although sweet rice wine is widely appreciated for its flavor and nutritional value, the traditional production method has limitations, including reliance on a single raw material, low fermentation efficiency, and insufficient diversity in product flavors. In recent years, to enhance the nutritional benefits and the flavor profiles of sweet rice wine, researchers have refined processing techniques and incorporated various ingredients [7,8]. Such advancements not only enrich the flavor of sweet rice wine but also endow it with additional health benefits.

Compared to polished rice, brown rice (BR) retains the bran and germ, making it a rich source of dietary fiber, vitamins, minerals, and other nutrients that positively influence human metabolism [9,10]. However, sweet rice wine made from BR has yet to appear in the market. The primary challenge lies in the physical barrier formed by the bran on the surface of BR, which hinders the ability of fermentation microorganisms, such as *Rhizopus oryzae*, or their secreted enzymes (e.g., amylases and proteases), to access the starch and proteins within the rice. This results in a lower wine yield [5]. Rice bran (RB), a major byproduct of polishing BR into white rice (WR), is produced in substantial quantities [11]. In China, annual RB production reaches 16.5–20.7 million tons. RB is not only rich in dietary fiber and other nutrients but also holds significant potential as a novel food ingredient [12]. Incorporating RB as an exogenous additive in sweet rice wine production could effectively address the issue of fermentation inhibition caused by the bran layer on BR. However, no studies have yet explored the effects of RB on the quality of sweet rice wine.

Extrusion is a commonly used food processing method and has been widely applied to the processing of whole grains such as BR [13]. This technology alters the physical structure and physicochemical properties of grains, making them more digestible and improving their taste and nutritional profile [14]. For instance, germinated BR extrudates, which are rich in bioactive components such as γ-aminobutyric acid, dietary fiber, and total ferulic acid, could enhance the palatability of bread [15]. Similarly, extrusion has been employed in the preparation of Chinese rice wine, where the pre-treatment of BR with extrusion improves the fermentation rate and saccharification efficiency [16]. Native RB is characterized by large particle size, high insoluble dietary fiber content, and uneven composition [17]. These factors may result in poor palatability when RB is directly added to sweet rice wine. Additionally, native RB is prone to rapid deterioration as a consequence of the hydrolytic degradation of lipids, thereby imposing limitations on its application as a foodstuff. Therefore, the second aim of this study is to apply extrusion to enhance the physicochemical properties of RB, thereby improving the quality of RB-enriched sweet rice wine.

In summary, this study first compared the effects of direct BR fermentation and fermentation with varying levels of RB addition on the wine yield, sugar-to-acid ratio, and flavor profile of sweet rice wine, aiming to optimize its production process. Building on this, the study further investigated how extrusion influences the microstructure and physicochemical properties of RB, elucidating the mechanisms by which extrusion improves the quality of RB-enriched sweet rice wine. The findings of this research provided theoretical insights and technical support for the development of whole-grain-based sweet rice wine products.

## 2. Materials and Methods

### 2.1. Materials and Reagents

Glutinous rice (Funuo 628), with starch content of 62.81 g/100 g, amylose content of 2.47 g/100 g, and protein content of 6.99 g/100 g, as well as BR and RB of the same variety, were provided by Huaiyuan Modern Agricultural Science and Technology Park (Bengbu, China) and harvested in 2022. Sweet rice wine starter (composed of rice flour and *Rhizopus oryzae*) was supplied by Angel Yeast Co., Ltd. (Yichang, China). All chemical reagents used in this experiment were of analytical grade and purchased from Sinopharm Chemical Reagent Co., Ltd. (Suzhou, China).

### 2.2. Rice Bran (RB) Extrusion

RB was mixed with 18% water using a mixer (Guangzhou Sanmai Machinery Equipment Co., Ltd., Guangzhou, China) at a speed of 350 r/min for 30 min. A twin-screw extrusion machine (DS32-VII, Jinan Saixin Machinery Co., Ltd., Jinan, China) was used to extrude the mixture at a feeding speed of 16 Hz and a screw speed of 24 Hz. During the extrusion process, the temperature settings were as follows: TP1 (50 °C, 80 °C, 100 °C, 110 °C), TP2 (50 °C, 80 °C, 100 °C, 120 °C), TP3 (50 °C, 80 °C, 110 °C, 130 °C), and TP4 (50 °C, 90 °C, 120 °C, 140 °C). The extruded products were dried at 40 °C, ground, and sieved through a 60-mesh screen for subsequent experiments.

### 2.3. Rice Wine Fermentation

Sweet rice wine was prepared according to previously reported method with a slight modification [5]. Glutinous rice (100.0 g) was washed three times, soaked at room temperature (25 °C) for about 8 h, and then the water was filtered. RB was then added at proportions of 0%, 2%, 4%, 6%, 8%, and 10% of the weight of the glutinous rice. An appropriate amount of water was added (rice:water = 1:0.8 (*w*/*v*)), and the mixture was cooked for 30 min. After cooling the samples to 25 °C, 0.4 g of sweet rice wine starter was added and the mixture was thoroughly mixed. The resulting mixture was fermented at 30 °C for 48 h. After fermentation, the mixture was filtered, heated at 80 °C for 10 min, then cooled and stored at 0–4 °C for further analysis. For the control, sweet rice wine was prepared using 100 g of BR under the same conditions. In subsequent experiments, RB processed by various extrusion treatments was added at a 4% (*w*/*w*) ratio and fermented under identical conditions.

### 2.4. Characterization of Extruded RB

#### 2.4.1. Basic Component Determination

The protein content was determined following the Kjeldahl method. Lipid content was measured using the Soxhlet extraction method. Soluble dietary fiber (SDF) and insoluble dietary fiber (IDF) were measured using the enzymatic–gravimetric method as described in AACC (2000) [18].

#### 2.4.2. Color Measurement

The color of the samples was measured using a colorimeter (CR-400, Konica Minolta, Tokyo, Japan), according to previously reported method [19]. The device was calibrated with a standard white ceramic plate, and then the color measurements were performed at three different locations. The values of L*, a*, and b* were recorded. Here, L* represents lightness (from dark to light), a* indicates the green (−) to red (+) spectrum, and b* represents the blue (−) to yellow (+) spectrum.

#### 2.4.3. Scanning Electron Microscopy (SEM)

The morphology of raw and extruded RB was observed using a scanning electron microscope (GeminiSEM300, Carl Zeiss Microscopy Co., Ltd., Oberkochen, Germany) at an acceleration voltage of 10 kV and magnifications of 100×, 1000×, and 5000× [20].

#### 2.4.4. Fourier-Transform Infrared (FT-IR) Spectroscopy

The effect of extrusion on the chemical structure of RB was determined using an FT-IR spectrometer (Antaris 2, Thermo Fisher Scientific, Waltham, MA, USA) [20,21]. RB samples were mixed with potassium bromide (KBr) at a ratio of 1:100 (*w*/*w*), thoroughly ground, pressed into 2 mm transparent pellets, and then scanned from 4000 cm^−1^ to 400 cm^−1^ with 48 scans.

#### 2.4.5. Water Solubility Index and Water Absorption Index Determination

RB (*W*, 1.00 g) was weighed and placed into a centrifuge tube with a total mass of *P*1. Water was added to adjust the volume to 50 mL. The mixture was shaken in a water bath at 25 °C for 30 min and then centrifuged at 4800 rpm for 20 min using a high-speed centrifuge (GL-20G-II, Anting Scientific Instrument Co., Ltd., Shanghai, China). The supernatant was carefully transferred to a pre-weighed aluminum dish and dried at 105 °C until constant weight was achieved, obtaining the mass of the dissolved powder (*A*). The mass of the centrifuge tube at this point was recorded as *P*2. The solubility (*S*) and water absorption rate (*B*) were calculated using the following formulas:

$$S(\%)=\frac{A}{W}\times 100$$

$$B(\%)=\frac{P2-P1}{W\left(100-S\right)}\times 100$$

### 2.5. Characterization of Sweet Rice Wine

#### 2.5.1. Determination of Physicochemical Properties

The alcohol content of sweet rice wine was measured following the method by Xu et al. [5] with slight modifications. Sweet rice wine (50 mL) was placed in a 500 mL distillation flask, and 100 mL of distilled water was added before starting the distillation. The distillation was stopped when the volume of the distillate reached 100 mL. Then, 1.0 mL of the distillate was placed in a test tube, and 0.5 mL of 0.0167 mol/L potassium dichromate solution and 2.0 mL of concentrated sulfuric acid were added sequentially. The mixture was thoroughly mixed and heated in a boiling water bath for 10 min. After cooling to room temperature, the absorbance was measured at a wavelength of 600 nm. A standard curve was prepared using ethanol as the standard substance. After the fermentation was completed, the liquid was filtered, and its volume was measured. The wine yield is expressed as the volume of liquid produced per 100 g of rice (mL/100 g). The total acid content of sweet rice wine was determined using the acid–base indicator titration method [8]. The sample was titrated using a standardized sodium hydroxide solution with phenolphthalein as the indicator. The reducing sugar content of sweet rice wine was measured using the 3,5-dinitrosalicylic acid (DNS) method [8]. Sweet rice wine (1 mL) was mixed with 2 mL of DNS reagent and reacted in a water bath at 80 °C for 5 min. After cooling to room temperature (~25 °C), the solution was diluted to 20 mL. The absorbance was measured at a wavelength of 540 nm. A glucose standard curve was prepared, and the results were expressed as the glucose equivalent per 100 g of the original sample (g/100 g).

#### 2.5.2. Antioxidant Activity Determination

The DPPH scavenging activity and ferric reducing antioxidant power (FRAP) were used as indicators to evaluate the antioxidant capacity of sweet rice wine [5]. Rice wine (200 μL) was mixed with 3.8 mL of 60 μmol/L DPPH-ethanol solution, and the mixture was reacted in the dark at 25 °C for 60 min in a water bath. The absorbance was measured at a wavelength of 517 nm and the DPPH radical scavenging activity was calculated. FRAP reagent was prepared by mixing 0.3 mol/L sodium acetate buffer, 10 mmol/L TPTZ solution, and 20 mmol/L FeCl_3_·6H_2_O solution in a 10:1:1 volume ratio. Then, 100 μL of rice wine was mixed with 3 mL of the FRAP reagent and 300 μL of deionized water, and the mixture was incubated in the dark at 25 °C for 30 min. The absorbance was measured at a wavelength of 593 nm. A standard curve was determined using ascorbic acid as the standard, and the results were expressed as the equivalent amount of vitamin C (μg/mL) per mL of sample.

#### 2.5.3. E-Tongue Analysis

The electronic tongue analysis of sweet rice wine was performed using the Insent taste sensor system (Insent Intelligent Sensor Technology Inc., Atsugi, Japan). The system was initially cleaned and self-calibrated before being used to assess sweetness, sourness, bitterness, astringency, saltiness, as well as aftertaste A, aftertaste B, and richness. The reference electrode and the five sensors (sourness CA0, bitterness C00, astringency AE1, umami AAE, and saltiness CT0) were activated for over 24 h, while the sweetness sensor (GL1) was pre-activated for at least 48 h before testing. Each sample was measured in triplicate.

#### 2.5.4. E-Nose Analysis

Sweet rice wine (3.0 g) was place into a headspace vial and then its odor characteristics were analyzed using the PEN 3 electronic nose system (Airsense Analytics Co., Ltd., Schwerin, Germany). After sealing the vial, it was incubated at 60 °C for 15 min. The test conditions were set as follows: the purge time was 120 s, the automatic zeroing time was 5 s, the sample preparation time was 5 s, the sampling interval was 1 s, the airflow rate during sampling and injection was 400 mL/min, and the measurement time was 120 s.

#### 2.5.5. Determination of Volatile Compounds

The volatile fingerprint of the sweet rice wine samples was analyzed using GC-IMS (Flavorspec^®^, Gesellschaft für Analytische Sensorsysteme mbH, Dortmund, Germany) equipped with an MXT-WAX chromatographic column (30 m × 0.53 mm × 0.1 μm, Restek, Shanghai, China) [22,23]. One milliliter of the sweet rice wine sample was placed in a 20 mL headspace vial and incubated at 50 °C for 10 min. After incubation, the headspace gas was injected into the injection port using a heated syringe (85 °C). The carrier gas was nitrogen (99.99% purity) with a flow rate of 1.0 mL/min. The chromatographic column temperature program was as follows: initial temperature at 60 °C, with a starting flow rate of 2 mL/min held for 2 min; the flow rate increased to 100 mL/min over 18 min; the flow rate of 100 mL/min was maintained for 20 min. All analyses were performed in triplicate. Volatile compounds were qualitatively identified using the GC × IMS Library Search 1.0.3 by comparing the retention index and drift times with the standards in the GC-IMS library.

### 2.6. Statistical Analysis

Data analysis was conducted using IBM SPSS Statistics 26 software, and all figures were drawn using Origin 2023 software. Data are expressed as the mean ± standard deviation of three replicate experiments. One-way analysis of variance (ANOVA) was performed, followed by Duncan’s multiple range test with a significance level of *p* < 0.05.

## 3. Results and Discussion

### 3.1. Effect of RB on Sweet Rice Wine Characteristics

#### 3.1.1. Alcohol Content and Wine Yield

**Figure 1:**
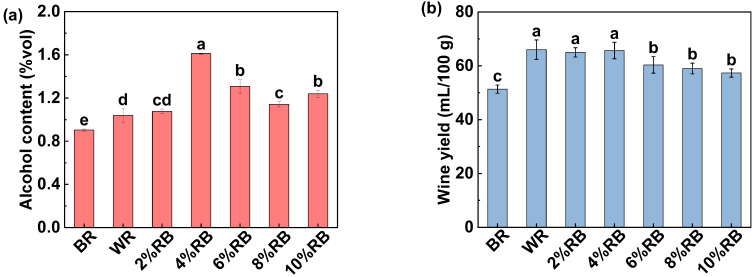

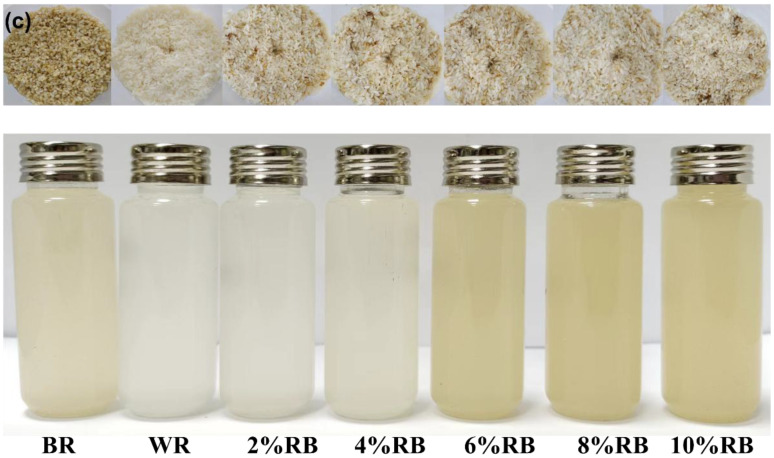
Effect of rice bran addition on the (**a**) alcohol content, (**b**) wine yield, and (**c**) appearance of rice wine. Note: BR represents brown rice wine; WR represents white rice wine; RB represents rice wine prepared with the addition of rice bran. Different letters indicate significant difference among treatments.

As shown in Figure 1, the BR wine exhibited the lowest alcohol content (Figure 1a) and wine yield (Figure 1b). The outer layer of BR reduces water absorption, resulting in insufficient gelatinization during cooking, and consequently, lower starch gelatinization and saccharification efficiency [24]. Additionally, the bran layer hinders nutrient transport, suppressing microbial growth and reducing fermentation efficiency, ultimately leading to lower alcohol production and wine yield. Xu et al. [5] also reported that BR wine exhibits a lower alcohol content than WR wine. The exogenous addition of RB effectively alleviated the interference caused by the outer layer of BR. As RB content increased, both the alcohol content and wine yield rose, peaking at 1.6% vol and 65.7%, respectively, when 4% RB was added. However, further increases led to a decline in alcohol content and wine yield (Figure 1a,b), likely due to the inhibitory effects of high fiber and lipid levels in RB on enzymatic activity and yeast metabolic efficiency. Notably, alcohol content in all groups remained below 2.0% vol, which was related with the characteristics of the sweet rice wine starter, which is dominated by *Rhizopus oryzae*. While this strain has strong saccharification abilities, it lacks efficient ethanol-producing capabilities [25], resulting in substantial sugar accumulation and relatively low alcohol yields. In summary, RB addition can mitigate the challenges of low alcohol content and wine yield in BR fermentation, and the amount of RB significantly influences fermentation characteristics.

#### 3.1.2. Appearance and Color

Figure 1c shows that the color of the BR wine was darker than that of WR group, 2% RB group, and 4% RB group, but lighter than that in the 6% RB, 8% RB, and 10% RB groups. Figure S1 confirms that the L* value of WR wine was highest, at 80.7, while the value decreased to 79.3 for BR wine. As the RB content increased from 2% to 10%, the L* value decreased from 79.9 to 76.2. Overall, the wine color deepened with increasing RB addition. Figure 1c also illustrates the states of wine lees in different groups. BR wine lees were relatively loose and easily dispersed when inverted, whereas WR wine lees and those from groups with added RB were more compact and remained intact when inverted. This suggests that, under identical soaking and cooking conditions, the gelatinization efficiency of BR was inferior to that of WR. The dense bran layer on BR reduced water absorption, impaired starch gelatinization [24,26], and subsequently hindered saccharification and fermentation, decreasing the cohesiveness between rice grains.

#### 3.1.3. Reducing Sugar Content and Total Acid Content

**Figure 2:**
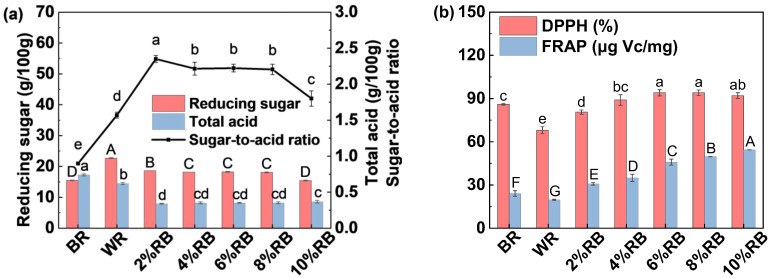
Effect of rice bran addition on the (**a**) reducing sugar content, total acid content, and sugar-to-acid ratio (solid line), and (**b**) DPPH scavenging activity and FRAP of rice wine. Note: Same as Figure 1. Different letters indicate significant difference among treatments.

Reducing sugar content, total acid content, and sugar-to-acid ratio are important indicators that reflect the sensory quality of sweet rice wine [22]. As shown in Figure 2a, the BR group exhibited the lowest reducing sugar content but the highest total acid content among all groups. Consequently, its sugar-to-acid ratio was significantly lower than that of other groups, adversely affecting its sensory characteristics. This difference may be attributed to the poor gelatinization efficiency of BR [24,26], which inhibits the subsequent saccharification and reduces the production of reducing sugars. Additionally, the lower saccharification efficiency in BR results in unconverted polysaccharides being metabolized by microorganisms during fermentation, leading to increased organic acid production [5]. Compared with BR wine, the addition of RB effectively improved the sugar-to-acid ratio in sweet wine. As the RB content increased from 2% to 10% (*w*/*w*), the reducing sugar content gradually declined, while the total acid content initially decreased but then slightly increased. Overall, moderate RB addition maintained the sugar-to-acid ratio at a relatively high level (>40), enhancing the sensory properties of rice wine.

#### 3.1.4. Antioxidant Capacity

As shown in Figure 2b, with the increase in RB content, the FRAP significantly improved, rising from 15.7 μg/mL to 43.6 μg/mL. The DPPH scavenging activity showed an initial increase followed by a slight decrease, rising from 68.0% to 94.0%, and then slightly decreasing to 92.0%. Therefore, as the RB content increased, the antioxidant capacity of the sweet wine generally improved. The FRAP of the BR group was significantly higher than that of the WR group but significantly lower than that of sweet wine with added RB. The DPPH scavenging activity of BR wine was comparable to that of sweet wine with 4% RB addition, but was lower than those adding 6–10% RB. This indicates that BR wine has strong antioxidant activity, and the addition of RB further enhances its antioxidant properties. Prior research noted that the improvement in antioxidant capacity is primarily related to the increased content of phenolic antioxidants during fermentation [5,27]. Schmidt et al. [28] found that fermentation with *Rhizopus oryzae* significantly increased the free phenolic content in RB. Yang et al. [1] suggested that during fermentation, the total phenolic content in rice wine significantly increased, likely due to microbial and enzymatic actions that facilitate the release of phenolic compounds from the rice.

### 3.2. Effect of Extrusion on the Quality of RB

#### 3.2.1. Appearance, Color and Morphology

**Figure 3:**
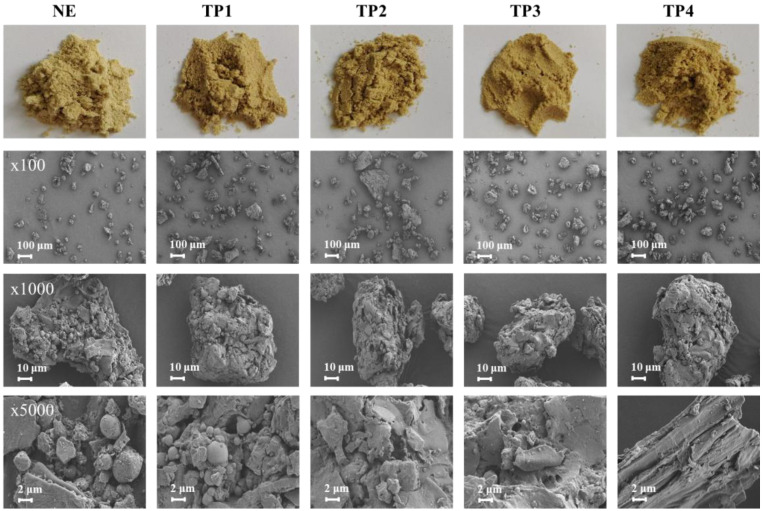
Effect of extrusion on the appearance and morphology of rice bran. Note: NE represents non-extruded rice bran; TP1: 50 °C, 80 °C, 100 °C, 110 °C; TP2: 50 °C, 80 °C, 100 °C, 120 °C; TP3: 50 °C, 80 °C, 110 °C, 130 °C; TP4: 50 °C, 90 °C, 120 °C, 140 °C.

**Figure 4:**
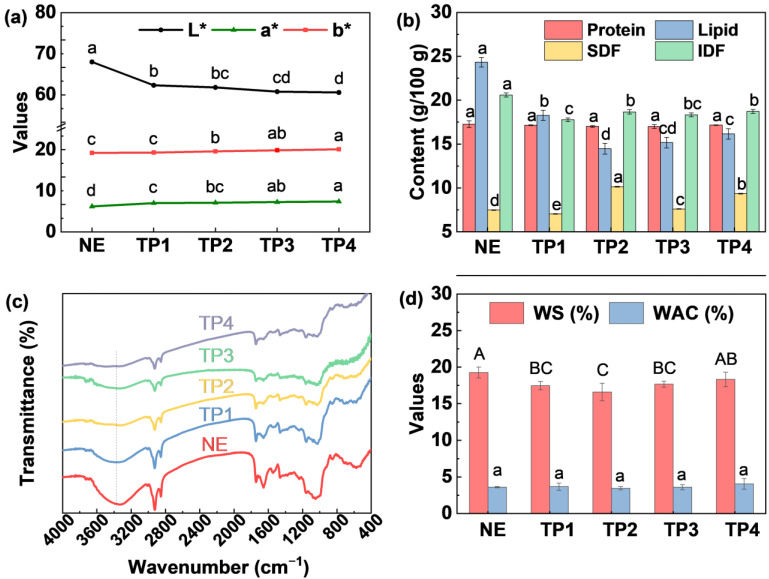
Effect of extrusion on the (**a**) color of rice bran, (**b**) basic component content, (**c**) chemical structure, (**d**) water solubility (WS) and water absorption capacity (WAC). Note: SDF and IDF represent the soluble dietary fiber and insoluble dietary fiber, respectively. Others are the same as Figure 3. Different letters indicate significant difference among treatments.

As shown in Figure 3, the color of RB progressively darkened with increasing extrusion temperature, exhibiting a tendency toward black and yellow. Figure 4a further illustrates that as the extrusion temperature increased, the a* and b* values exhibited an upward trend, while the L* value showed a downward trend, indicating a darkening of the RB color. This may be attributed to the Maillard reaction during extrusion [19]. The microstructure of non-extruded RB (NE) and RB subjected to different extrusion procedures (TP1, TP2, TP3, TP4) was observed using SEM (Figure 3). NE exhibited a uniform particle size distribution, with clearly identifiable spherical starch granules. Extrusion altered the particle morphology and structure of the RB. With increasing extrusion temperature, the spherical structures were progressively disrupted, and linear fibrous structures became increasingly exposed. These changes are attributed to the high temperature, pressure, and shear forces during the extrusion process [29]. The disruption of the fibrous structure in RB may enhance its fermentation characteristics, which will be further discussed in Section 3.3.

#### 3.2.2. Basic Component Content

Extrusion had no significant effect on the protein content of RB, with the protein content of all groups remaining stable at 17.0–17.3 g/100 g (Figure 4b). However, extrusion significantly reduced the lipid content of RB, a modification that is beneficial for enhancing its storage stability. Rashid et al. [30] reported that the fatty acid contents in extruded RB remained much lower than the control during storage. Dietary fiber, a key functional component of RB, is typically categorized into soluble dietary fiber (SDF) and insoluble dietary fiber (IDF) based on water solubility [21]. During extrusion, the temperature raised rapidly, accompanied by high pressure and shear forces, leading to the cleavage of glycosidic bonds in fiber polymers and the formation of numerous hydrophilic groups [29]. This transformation partially degraded dietary fiber, generating smaller, more soluble compounds, thereby increasing the proportion of SDF [21]. In this study, TP2, TP3 and TP4 resulted in an increase in SDF content and a decrease in IDF content.

#### 3.2.3. FT-IR Spectra

The effects of extrusion on the chemical structure of RB were analyzed using FT-IR (Figure 4c). The peak observed between 3600 and 3200 cm^−1^ corresponds to O-H stretching vibrations [16,21]. The NE sample exhibited higher peak intensity, whereas the extruded samples (TP1–TP4) showed progressively weaker intensities, indicating a reduction in hydroxyl group content. This reduction is likely due to the disruption of hydrogen bond interactions among biomacromolecules in RB [29]. The peak near 1730 cm^−1^ corresponds to C=O stretching vibrations, characteristic of esters, carboxylic acids, and aldehydes [21]. While the NE sample displayed a prominent peak in this region, its intensity gradually weakened in the extruded samples, possibly reflecting the breakdown of ester bonds and partial lipid degradation. The peak between 1600 and 1500 cm^−1^ represents C=C stretching vibrations in aromatic rings. The extruded samples exhibited a gradual decrease in peak intensity in this region, suggesting structural changes in polyphenolic compounds. The region between 1300 and 1000 cm^−1^ corresponds to C-O-C and C-O stretching vibrations [21]. Distinct peaks were observed in the NE sample, but their intensity progressively declined in the extruded samples, indicating that extrusion may disrupt polysaccharide structures, such as cellulose and hemicellulose. Overall, the most significant changes were observed in the 3600–3200 cm^−1^ and 1200–1000 cm^−1^ regions, highlighting that extrusion significantly affected hydrogen bonds and polysaccharide structures, which may contribute to the improved functionality of RB. Additionally, the substantial changes near 1730 cm^−1^ underscore the impact of extrusion on lipid degradation, consistent with the results shown in Figure 4b.

#### 3.2.4. Water Solubility Index and Water Absorption Index

Qiao et al. [21] reported that high-temperature extrusion may break polymer chains, resulting in increased water solubility. However, in this study, a slight decrease in water solubility of RB was observed (Figure 4d), which might be attributed to the more compact structure due to high pressure treatment during extrusion. Meanwhile, the water absorption capacity of all samples remained below 5%, with no significant differences observed between the treatment groups (Figure 4d).

### 3.3. Effect of Extruded RB on Sweet Rice Wine Characteristics

#### 3.3.1. Color, Alcohol Content, Wine Yield and In Vitro Antioxidant Capacity

**Figure 5:**
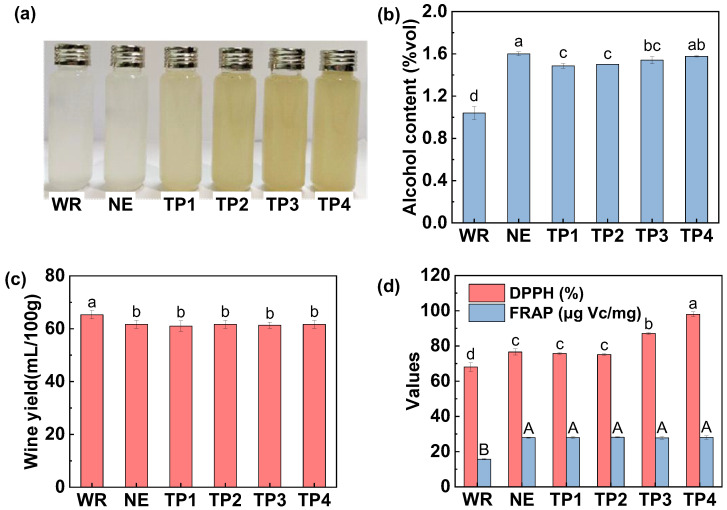
Effect of extrusion on the (**a**) appearance, (**b**) alcohol content, (**c**) wine yield, and (**d**) antioxidant capacity of rice bran-enriched rice wine. Note: WR represents rice wine prepared using white rice as raw material. Others were the same as Figure 3. Different letters indicate significant difference among treatments.

Sweet rice wine made from WR as raw materials boasts excellent taste and is highly favored by consumers [7]. Therefore, this study compared the quality differences between rice wine prepared with the addition of extruded BR and WR wine, highlighting the potential application of extrusion treatment in improving the quality of BR wine. Figure 5a shows that the WR and NE groups had a similar milky white color, as indicated by comparable a*, b* and L* values. However, after extrusion, the color of the sweet rice wine became noticeably more yellow and darker, as evidenced by decreased L* and a* values and an increased b* value, which is consistent with the results reported in Figure 4a. Figure 5b indicates that the alcohol content of the NE group and the extruded RB groups (TP1, TP2, TP3, TP4) was significantly higher than that of the WR group, with alcohol content ranging from 1.4 to 1.7% vol. Figure 5c reveals that the wine yield of the WR group was significantly higher than that of the other groups, while no significant differences were observed in wine yield among the remaining groups. This suggests that, compared to untreated RB, extrusion had no significant effect on the wine yield of sweet rice wine. Although Kuo et al. [16] reported that extrusion can promote the fermentation of BR, this effect is attributed to the pre-gelatinization of starch in the BR induced by extrusion.

Figure 5d shows that compared with NE, the DPPH radical scavenging capacity of sweet rice wine significantly increased from 76.6% to 98.0% with extrusion, while the FRAP remained unchanged for the NE, TP1, TP2, TP3, and TP4. Phenolic compounds play a key role in antioxidant activity. RB is rich in phenolic compounds [11], while the high temperature and pressure during extrusion likely damaged the cell wall structure, facilitating the release of bound phenolic compounds, thereby increasing the antioxidant activity of the wine [31]. Notably, both the DPPH radical scavenging capacity and FRAP of NE, TP1, TP2, TP3, and TP4 groups were significantly higher than WR wine, suggesting that the addition of RB could significantly increase the in vitro antioxidant capacity of sweet rice wine.

#### 3.3.2. Electronic Tongue and Electronic Nose Results

**Figure 6:**
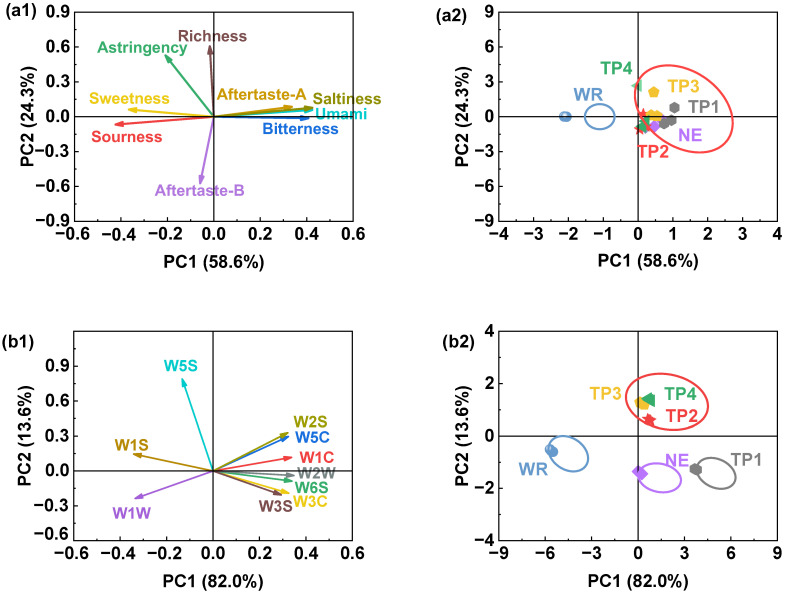
Effect of extrusion on the (**a1**) loading plot and (**a2**) score plot of E-tongue, (**b1**) loading plot and (**b2**) score plot of E-nose of rice bran-enriched rice wine. Note: Same as Figure 5.

In the electronic tongue analysis, the potential values of each sensor reflect the intensity of the corresponding taste. Principal component analysis (PCA) revealed that PC1 explained 58.6% of the variance, while PC2 accounted for 24.3%, with a cumulative contribution of 82.9%, capturing the majority (>80%) of the taste information from the samples (Figure 6(a1)). The score plot (Figure 6(a2)) shows that the six sweet rice wine samples were distributed into two main regions, with the WR group being distinctly separated from the other groups. Tang et al. [22] also reported a significant difference in taste for black glutinous rice wine and white glutinous rice wine. The NE group exhibited overlap with the TP1, TP2, TP3, and TP4 groups, indicating that these groups shared similar taste characteristics. Combining these findings with the loading plot (Figure 6(a1)), it is apparent that sourness and sweetness were the predominant contributors to the overall flavor profile of the WR group [5], whereas aftertaste A, saltiness, umami, and bitterness played more prominent roles in the flavor profiles of the other groups.

PCA of the electronic nose data revealed that PC1 explained 82.0% of the variance, and PC2 accounted for 13.6%, with a cumulative contribution of 95.6%, effectively capturing the majority of the odor information from the samples (Figure 6(b1)) [5]. The score plot (Figure 6(b2)) shows that the WR, NE, and TP1 groups formed distinct, non-overlapping clusters, suggesting that their odor profiles were sufficiently different to be distinguished by the electronic nose. In contrast, the TP2, TP3, and TP4 groups exhibited overlapping distributions, indicating that these groups shared similar odor characteristics and were indistinguishable to the electronic nose. The WR group was positioned in the negative direction along the horizontal axis, and the loading plot (Figure 6(b1)) indicated that W1S, W1W, and W5S were the major contributors to its odor. These components are primarily associated with nitrogen oxides and inorganic sulfur compounds, which align with the characteristic aroma of sweet rice wine. In comparison, the NE, TP1, TP2, TP3, and TP4 groups exhibited distributions closer to W1C, W3C, W5C, and W2S, indicating that their odor profiles were more strongly associated with aromatic compounds, alkenes, alkanes, and alcohols. Notably, W2S represents alcohol-based aromatic compounds, and the TP1, TP2, TP3, and TP4 groups showed a closer alignment with W2S than the NE group. These findings suggest that the addition of extruded RB enhances the alcohol-like aroma of sweet rice wine, consistent with the alcohol content results (Figure 1a).

#### 3.3.3. Volatile Compounds

**Figure 7:**
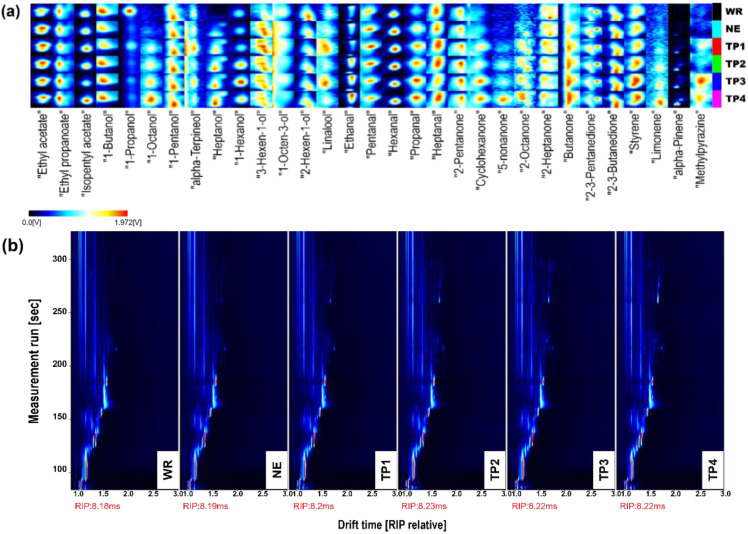
(**a**) Fingerprint of volatile compounds and (**b**) 2D-topographic plots of volatile compounds of rice bran-enriched rice wine.

As shown in Figure 7a,b, a total of 31 volatile flavor compounds were detected in the six sweet rice wine samples by GC-IMS, including three esters, eleven alcohols, five aldehydes, eight ketones, and four other compounds. Ester compounds contribute fruity or distinctive floral aromas to sweet rice wine and are key flavor-active substances [12]. Alcohols primarily define the alcoholic aroma of the wine, while aldehydes and ketones enhance both the aroma and mouthfeel [22]. Compared to the WR group, the NE, TP1, TP2, TP3, and TP4 groups exhibited characteristic compounds such as 1-octanol, heptanol, 5-nonanone, 2-octanone, and butanone, indicating that the addition of RB enriched the volatile flavor compound profile. Extrusion has a complex effect on the release and transformation of flavor compounds in RB. It likely disrupts the cell wall structure of RB due to high temperature and shear force, facilitating the release of endogenous nutrients and creating more favorable conditions for microbial metabolism [29]. Overall, the addition of RB and extrusion increased the content of aromatic compounds in sweet rice wine, thereby enhancing its flavor complexity and overall quality.

## 4. Conclusions

**Figure 8:**
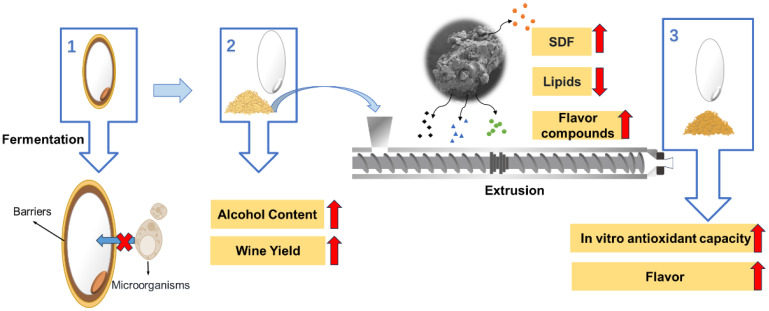
Possible mechanism of rice bran affecting fermentation and extrusion improving the quality of rice bran-enriched rice wine. 1. Direct fermentation of brown rice. 2. Fermentation of the mixture of white rice and rice bran. 3. Fermentation of the mixture of white rice and extruded rice bran. Note: SDF means soluble dietary fiber.

This study successfully improved the quality of BR wine by exogenously adding RB and extrusion. The bran layer on the surface of BR hinders the contact between the rice wine starter and the nutrients in rice such as starch, thereby inhibiting the fermentation process. As a result, wine produced from BR as raw materials exhibits higher acidity and inferior taste. The exogenous addition of RB can effectively improve the fermentation process, yielding rice wine with improved alcohol content, a suitable sweet-to-acid ratio, and improved antioxidant capacity. Extrusion effectively disrupted the granular structure of RB, promoting the release of endogenous functional substances, further enhancing the fermentation process and boosting the antioxidant capacity of the resulting rice wine. In addition, the high temperature and pressure during the extrusion process may induce Maillard reactions and caramelization, leading to browning of the RB’s color and alterations in its flavor. The possible mechanism by which adding RB and extrusion improved the quality of BR wine is shown in Figure 8. In conclusion, this study demonstrates that the exogenous addition of rice bran combined with extrusion treatment can significantly enhance the quality of rice wine, providing a novel approach for the development of innovative rice wine products by producers. However, it is worth noting that extrusion treatment may cause browning of the rice wine. In the future, additional technologies such as ultrafine grinding could be employed to further enhance product quality.

## Data Availability

The original contributions presented in this study are included in the article/Supplementary Materials. Further inquiries can be directed to the corresponding author.

## Supplementary Materials

The following supporting information can be downloaded at: https://www.mdpi.com/article/10.3390/foods14091582/s1, Figure S1: Effect of rice bran addition on the color of rice wine; Figure S2: Effect of extrusion on the color of rice bran-enriched rice wine; Figure S3: Raw picture for the appearance and morphology of rice bran subjected to extrusion. Note: NE represents non-extruded rice bran; TP1: 50 °C, 80 °C, 100 °C, 110 °C; TP2: 50 °C, 80 °C, 100 °C, 120 °C; TP3: 50 °C, 80 °C, 110 °C, 130 °C; TP4: 50 °C, 90 °C, 120 °C, 140 °C.
