# Supplementary material for: Preparation of Rice Bran-Enriched Sweet Rice Wine and Its Quality Improvement Through Extrusion
Source: Foods. 2025 Apr 30;14(9):1582. doi: 10.3390/foods14091582 (PMC12071912; doi:10.3390/foods14091582)
Supplement: Supplementary file 1 [file foods-14-01582-s001.zip › foods-3581922-supplementary.pdf]

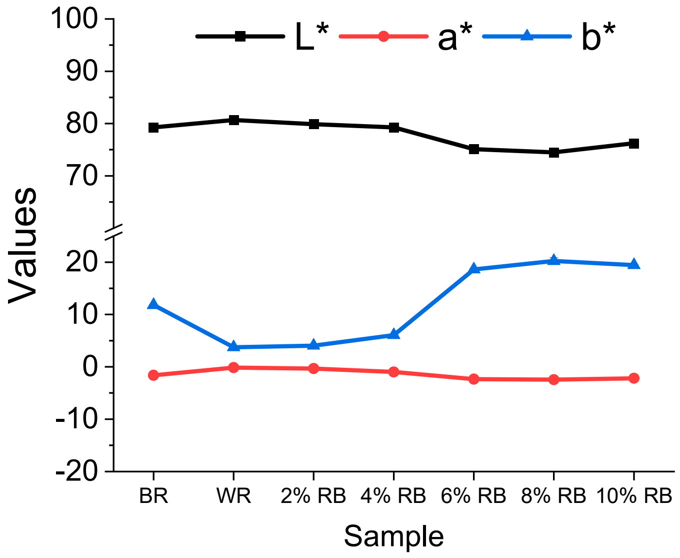

Figure S1. Effect of rice bran addition on the color of rice wine.

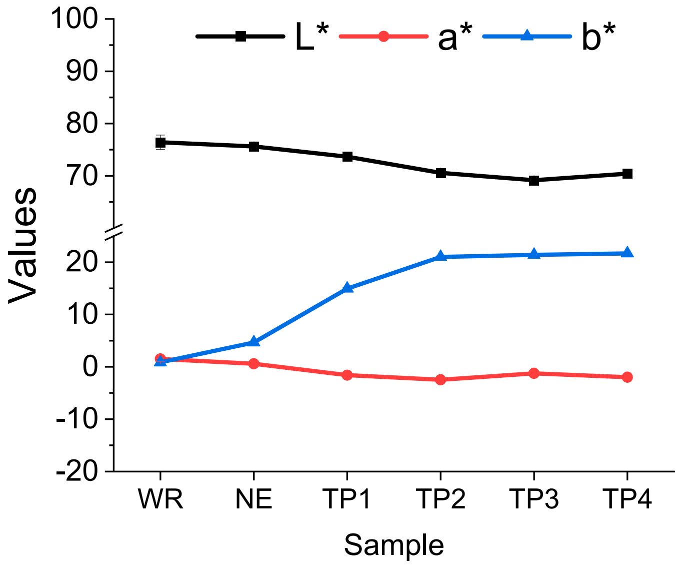

Figure S2. Effect of extrusion on the color of rice bran-enriched rice wine.

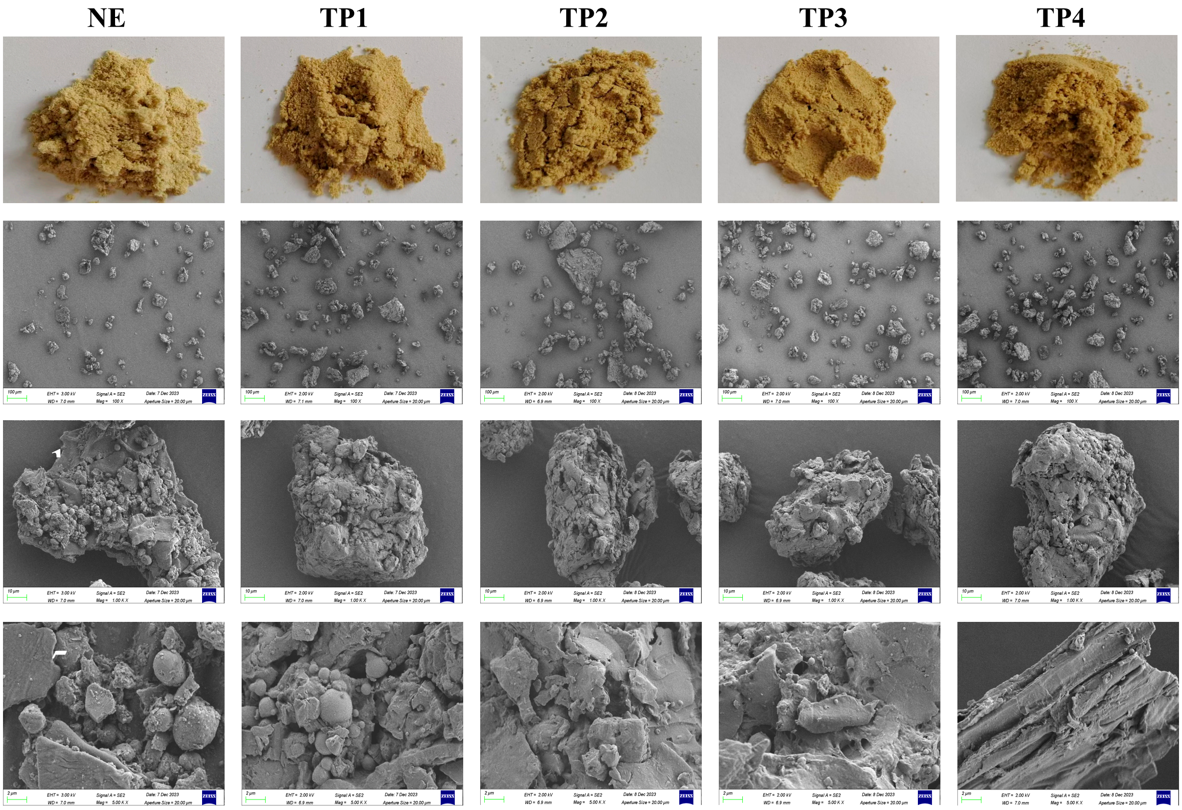

Figure S3. Raw picture for the appearance and morphology of rice bran subjected to extrusion. Note: NE represents non-extruded rice bran; TP1: 50°C, 80°C, 100°C, 110°C; TP2: 50°C, 80°C, 100°C, 120°C; TP3: 50°C, 80°C, 110°C, 130°C; TP4: 50°C, 90°C, 120°C, 140°C.
